# Role of Functional Biomarkers to Identify Early Vitamin B12 Deficiency in Patients with Sleeve Gastrectomy: A Cross-Sectional Study

**DOI:** 10.3390/medicina56030142

**Published:** 2020-03-20

**Authors:** Osama Y. Al-Dirbashi, Charu Sharma, Nahid Al Dahouri, Anas Al Aidaros, Shamma Al-Muhairi, Rami Beiram, Salah Gariballa, Juma Al Kaabi

**Affiliations:** 1 Department of Pediatrics, College of Medicine and Health Sciences, United Arab Emirates University, Al Ain 17666, UAE; dirbashios@uaeu.ac.ae (O.Y.A.-D.); nahidali@uaeu.ac.ae (N.A.D.); a.aidaros@uaeu.ac.ae (A.A.A.); 2 Department of Internal Medicine, College of Medicine and Health Sciences, United Arab Emirates University, Al Ain 17666, UAE; charusharma@uaeu.ac.ae (C.S.); s.gariballa@uaeu.ac.ae (S.G.); 3 Ambulatory Medicine (Dep) - Medical Affairs, Tawam Hospital (S.A.M.), Al Ain 17666, UAE; shmuhairi@seha.ae; 4 Department of Pharmacology, College of Medicine and Health Sciences, United Arab Emirates University, Al Ain 17666, UAE; rbeiram@uaeu.ac.ae

**Keywords:** bariatric surgery, homocysteine, methylmalonic acid, methylcitric acid, vitamin B12, UAE

## Abstract

*Background and objectives:* Although laparoscopic sleeve gastrectomy (LSG) is effective for obesity management, postoperative vitamin B12 (B12) deficiency is of major concern. In this cross-sectional study, we assessed the levels of B12 and its related functional biomarkers, namely, total homocysteine (tHcy), methylmalonic acid (MMA), folate, methylcitric acid (MCA), and hemoglobin (Hb), in one-year postoperative LSG patients and matched controls. *Materials and Methods:* Plasma B12, tHcy, MMA, folate, and MCA were measured in matched controls (n = 66) and patients (n = 71) using validated liquid chromatography-tandem mass spectrometry techniques and protocols in the United Arab Emirates (UAE). *Results:* The median B12 concentration in patients (177 pmol/L) was significantly lower (*p* < 0.001) than in the controls (334.7 pmol/L). The tHcy and MMA levels were significantly increased (*p* < 0.001 and *p* = 0.011, respectively) and folate levels were significantly decreased (*p* = 0.001) in the LSG patients compared to the controls. Interestingly, no significant difference in MCA levels were observed between the two groups. The levels of tHcy and MMA were concomitantly increased with the decreased folate levels in postoperative LSG patients when compared with the controls. The Hb levels were significantly lower in males and females in the patient group compared with those in the control group, respectively (*p* = 0.005 and *p* = 0.043). *Conclusions:* This is the first report of serum levels of B12 and its functional biomarkers in postoperative LSG patients among a local population from the UAE. Our findings revealed significant alterations of the B12 biomarkers, total B12, MMA, and tHcy in one-year postoperative LSG patients.

## 1. Introduction

The United Arab Emirates (UAE) has one of the highest obesity rates [1]. Despite various strategies, including lifestyle interventions and drug therapies, managing obesity remains a therapeutic enigma. In recent years, surgical interventions have garnered increasing attention, and bariatric surgery is emerging as one of the most effective treatments for severely obese people [2,3].

Among the available surgical options, laparoscopic sleeve gastrectomy (LSG) is a safe and effective procedure to manage morbid obesity [4,5]. LSG is used as a stand-alone procedure with good outcomes and is recommended as an alternative technique to the laparoscopic Roux-en-Y gastric bypass (LRYGB) [6,7]. Despite its clinical success and wide acceptance, a large proportion of LSG patients experience postoperative gastrointestinal (GI) side effects, including macronutrient and micronutrient deficiencies or an aggravation of previous nutritional deficits [8,9,10,11]. Furthermore, the structural and functional GI alterations of LSG are associated with the risk of developing vitamin B12 (B12) deficiency, mainly cobalamin and cyanocobalamin [12,13].

B12 is an indispensable water-soluble micronutrient that occurs in several forms, including cyano-, hydro-, methyl-, and 5′-deoxyadenosylcobalamin. Traces of cyanocobalamin occur in food items, but it is predominantly used in vitamin pills and fin fortified food. All forms of B12 are converted to methylcobalamin and 5′-deoxyadenosylcobalamin in the body, the forms that serve as coenzymes for B12. The other forms of cobalamin are interconvertible by the enzymes methylmalonyl-coenzyme A (CoA) mutase and methionine synthase (MS) [14]. Methylmalonyl-CoA mutase converts methylmalonyl-CoA to succinyl-CoA, with 5-deoxy adenosylcobalamin required as a cofactor. MS catalyzes the methylcobalamin-dependent (re)methylation of total homocysteine (tHcy) to the essential amino acid methionine within the methionine cycle. This reaction is folate-dependent, with the methyl group of methyltetrahydrofolate being transferred to tHcy to form methionine and tetrahydrofolate. MS disturbances lead to disruptions in the intracellular folate pathway. B12 deficiency leads to an impairment in these enzymes, resulting in the accumulation of their substrates, namely, methylmalonic acid (MMA) and tHcy [15,16,17].

In patients undergoing bariatric surgery the binding of B12 to intrinsic factor (IF), which is required for B12 absorption, is largely reduced. Although low serum levels of this vitamin in support with clinical evidence may indicate a deficiency, this approach is unreliable because most patients with a subclinical disease have normal B12 levels [18,19,20,21]. To date, there is no single test that can accurately detect B12 deficiency [18,19,20,21]. Previous studies have confirmed that serum B12 is of limited diagnostic value as a stand-alone biomarker of this condition [18,19,20,21]. The estimation of MMA and tHcy, the substrates that require B12 for correct metabolism, is arguably a better predictor, as these markers are sensitive to changes in B12 concentrations and may accumulate even before the serum level of this vitamin falls below the normal range [22].

Methylcitric acid (MCA) is a specific metabolite that accumulates in patients with B12 defects because of the condensation of oxaloacetate in the Krebs cycle with the accumulating propionyl-CoA. Although, elevated MCA levels associated with B12 metabolic defects have been reported. However, a determination of MCA in biological samples is still generally uncommon, mainly due to its challenging analytical properties, such as high polarity, lack of chromophore or fluorophore, and its presence in an enantiomer form [23,24,25,26,27,28]. Therefore, the present study was carried out to investigate whether MCA could be used as biomarker for B12 deficiency in LSG patients. In the present study, we determined the levels of B12 along with its functional biomarkers in postoperative LSG patients and compared the results with matched control subjects selected based on their likelihood to undergo LSG in the future.

## 2. Materials and Methods

### 2.1. Subjects

We enrolled study participants from the Obesity Center at Tawam Hospital, UAE, from March 2016 to May 2017. Tawam Hospital located in Al Ain City in the UAE is a 700-bed inpatient facility that includes over 92 specialty clinics and houses a comprehensive Obesity Center consisting of 4 bariatric surgeons, 3 family physicians, 1 endocrinologist, 1 pediatrician, 3 dieticians, 1 psychologist, and 2 care coordinators. This center deals with more than 150 patients a day and performs over 1000 bariatric surgeries annually. We included patients in our study if they had undergone LSG surgery with an at least 12-month postoperative period (n = 71) at the Tawam Hospital Obesity Center. Body weight, height, and body mass index (BMI) were recorded for anthropometric evaluation. All control group participants were identified as prospective candidates for bariatric surgery (n = 66) at the Obesity Center at Tawam Hospital and were matched for age, gender, and BMI. Prospective controls with a past or current history of substance abuse, psychiatric illness, endocrine or other disorders that can cause obesity, preoperative gut malabsorption syndrome, the presence of any gastric, kidney, and liver disease, or the preoperative use of medication affecting bone metabolism were excluded (Table 1). This cross-sectional study was approved by the Al Ain District Human Research Ethics Committee (#16/008 2016-472 with date of approval 26 February 2016) and was conducted in accordance with the Declaration of Helsinki. Written informed consent was obtained from all participants who expressed their interest in participating and were well informed of the study objectives.

### 2.2. Biomarker Measurements

The plasma levels of tHcy, MMA, and MCA were measured using a liquid chromatography (LC)-tandem mass spectrometry (MS/MS) system. B12, folate, and hemoglobin (Hb) levels were measured using a cobas e 411 modular analyzer (Roche Diagnostics, Indianapolis, IN, USA) with standard kits for B12, folate, and Hb estimation (Roche Diagnostics).

MMA, tHcy, MCA, and deuterium-labeled Hcy (d4-Hcy) were purchased from Sigma Aldrich (Taufkirchen, Germany). Deuterium-labeled MCA (d3-MCA) and deuterium-labeled MMA (d3-MMA) were used as internal standards and were purchased from Cambridge Isotopes Laboratories (Tewksbury, MA, USA). 4-[2-(N, N-dimethylamino) ethylsulfamoyl]-7-(2-amino ethyl amino) benzofurazan (DAABD-AE), 1-ethyl-3-(3-dimethylaminopropyl) carbodiimide (EDC), 4-(dimethyl-amino) pyridine (DMAP), perfluorooctanoic acid (PFOA), and formic acid were obtained from Sigma Aldrich. 3N HCl in *n*-butanol was purchased from Regis Technologies Inc. (Morton Grove, IL, USA). LC-MS/MS-grade acetonitrile, double distilled H_2_O, and methanol were purchased from Merck (Darmstadt, Germany).

Chromatographic analysis was performed using a Shimadzu Nexera X2 ultra high-performance liquid chromatograph system composed of 2 solvent delivery pumps and a thermostat-controlled autosampler, column oven, degasser, and system controller (Shimadzu Corporation, Kyoto, Japan). An LC-MS-8060 triple quadrupole mass spectrometer equipped with an electrospray ionization (ESI) source was used as the detector (Shimadzu Corporation, Kyoto, Japan).

### 2.3. Sample Preparation for LC-MS/MS

#### 2.3.1. tHcy Estimation

Briefly, 10 μL of plasma was added to 100 μL of 2.4 µmol/L d4-Hcy and 20 μL of 500 mmol/L 4-dithiothreitol. The mixture was vortexed for 30 s and centrifuged at 10,000 rpm for 3 min. The supernatant was transferred to a glass tube and dried using a stream of nitrogen at 45 °C for 5 min. Then, 50 μL of butanolic HCl was added and incubated for 30 min at 65 °C, followed by evaporation to dryness using nitrogen at 45 °C. Following sample reconstitution in 400 μL of 50% acetonitrile containing 10% formic acid, 2 μL was injected into the LC-MS/MS system. A calibration curve was prepared using plasma spiked with tHcy over a range of 0.5–100 μmol/L. Two tHcy quality control (QC) samples (7.35 μmol/L and 14.7 μmol/L) were used. LC separation using an Acquity BEH C18 column (2.1 × 50 mm, 1.7 μm; Waters, Milford, MA, USA) was achieved isocratically using 50% acetonitrile containing 0.05% PFOA at a flow rate of 0.4 mL/min and a total run time of 1.0 min (Figure S1). The ESI-MS/MS analysis was in positive ion mode with an interface voltage of 4.0 kV, desolvation temperature of 250 °C, ion source temperature of 300 °C, and collision energy of −15 eV using argon as the collision gas. Transitions of *m/z* 192.0 to *m/z* 90.1 and *m/z* 196.0 to *m/z* 94.0 were used to monitor tHcy and d4-tHcy, respectively.

#### 2.3.2. MMA Estimation

Briefly, 10 µL of plasma was added to 100 µL of 1.0 µmol/L of d3-MMA, vortexed for 1 min, and centrifuged at 10,000 rpm for 3 min. The supernatant (100 µL) was transferred to a glass tube and evaporated using a stream of nitrogen for 3 min. Then, 50 µL of 3N HCl in *n*-butanol was added to the residue and incubated for 15 min at 65 °C. After evaporating the excess butanolic HCl using a stream of nitrogen, the residue was reconstituted in 300 µL of 50% acetonitrile, and 20 µL was injected into the LC-MS/MS system. A calibration curve was prepared using plasma spiked with MMA over a range of 0.78–25.0 μmol/L. Two MMA QC samples (9.7 μmol/L and 20.0 μmol/L) were used. LC separation using an Acquity BEH C18 column (2.1 × 50 mm, 1.7 μm; Waters) was achieved isocratically using 50% acetonitrile containing 0.1% formic acid at a flow rate of 0.6 mL/min and a total run time of 3.3 min (Figure S2). The MS/MS analysis was in positive ion mode with an interface voltage of 4.0 kV, desolvation temperature of 250 °C, ion source temperature of 300 °C, and collision energy of 12 eV using argon as the collision gas. Transitions of *m/z* 231.2 to *m/z* 119.2 and *m/z* 234.2 to *m/z* 122.2 were used to monitor MMA and MMA-d3, respectively.

#### 2.3.3. MCA Estimation

Briefly, 20 µL of plasma was added to 40 μL of 2.4 μmol/L d3-MCA and 10 μL of acetonitrile. The mixture was vortexed for 1 min and centrifuged for 4 min at 3000 rpm. Then, 50 µL of the supernatant was transferred to a 1.5 mL microcentrifuge tube, followed by the addition of 100 μL of a 1:1:2 (v/v/v) mixture of EDC (25 mmol/L in H_2_O), DMAP (25 mmol/L in acetonitrile), and DAABD-AE (2 mmol/L in 90% acetonitrile). The sample mixture was vortexed and incubated for 45 min at 65 °C. The reaction was stopped with the addition of 900 µL of 10% methanol containing 0.05% PFOA, and 2 µL of the resultant mixture was injected into the LC-MS/MS system. The total run time was 8.10 min, and the MCA peak appeared at 3.67 min (Figure S3). A calibration curve was prepared using plasma spiked with MCA over a range of 0.25–10 μmol/L. Two MCA QC samples (3.75 μmol/L and 10 μmol/L) were used. The mass spectrometric analysis was in positive ion mode with an interface voltage of 4.0 kV, desolvation temperature of 250 °C, ion source temperature of 300 °C, and collision energy of 25 eV using argon as the collision gas. Transitions of *m/z* 499.0 to *m/z* 151.1 and *m/z* 502.0 to *m/z* 151.1 were used to monitor MCA and d3-MCA, respectively [29,30] 

### 2.4. Statistical Analysis

The data were analyzed using the SPSS Statistics v25 software (IBM Corp., Armonk, NY, USA). We ran the Q–Q and P–P plot to assess the normality distribution of B12, MMA, and tHcy. Because of highly skewed distributions of all, non-parametric Mann–Whitney were used in medians for the comparison of these parameters to describe patient demographics and differences in the levels of biomarkers between groups. The data are presented as median (range) and all test results were considered statistically significant when the two-tailed *p*-values were <0.05.

## 3. Results

### 3.1. Demographics and Medication Profile in Patients One Year after LSG

The demographic characteristics of the patients and controls are presented in Table 1. The details of diseases present in the patients were as follows: thyroid disease, one; stroke, one; depression, two; acanthosis nigricans, two; fatty liver, seven; and arthropathy, eleven. The number of smokers were ten. The details of patients who were taking medication were as follows: B12, eight; neurobion, two; vitamin D, twenty-one; multivitamins, five; a combination of calcium and vitamin D, five; proton pump inhibitors, seven; iron, nine; and zinc, one. None of the patients were taking aspirin or thiamine supplements.

### 3.2. B12, tHcy, and MMA in Patients and Controls

The median concentrations of B12, tHcy, MMA, and other biomarkers in patients and controls are shown in Table 2.

There was a significant difference (*p* < 0.001) in median B12 levels between the control and patient groups (334.7 pmol/L and 177.0 pmol/L, respectively) (Table 2). The majority of patients (61%) had suboptimal B12 levels, with only 28% showing <148pmol/L B12 levels (*p* < 0.001). In the control group, there were only 5% cases with low B12 levels (Table 3). A statistically significant increase in the levels of tHcy (*p <* 0.001) and MMA (*p* = 0.011) was detected in patients compared with the controls. tHcy levels were elevated in 55% of patients compared with 18% of controls (*p* = 0.001). Elevated MMA levels were found in 28% of patients compared with 18% of controls.

### 3.3. Folate Levels in Patients and Controls

Although median plasma folate levels fell within the optimal range (>9 nmol/L), we still found a significant difference in folate levels in patients (11.15 nmol/L) than in controls (22.4 µmol/L) (*p* < 0.001) (Table 2). While all participants in the control group had folate levels >9 nmol/L, 30% of patients had low levels of folate (*p* < 0.001).

### 3.4. MCA Levels in Patients and Controls

There was no statistically significant difference in median MCA levels between the control and patient groups (*p* = 0.266) (Table 2). There were only two patients who showed elevated MCA levels (Table 3). Typical chromatograms from the patient and control groups obtained using LC-MS/MS for MCA analysis are shown Figure S3.

### 3.5. Hb Levels in Patients and Controls

Hb levels were not found significantly different among the patients and controls. However, when analyzed on basis of gender, there was a significant difference in Hb levels in both males and females in the patient and control groups (*p =* 0.005 and *p* = 0.043) (Table 2).

### 3.6. Comparison of Metabolite Levels with B12 Levels in Patients and Controls using Different Approaches

There was a statistically significant difference in patients and controls who had both deficient B12 and elevated tHcy levels (*p =* 0.057). Moreover, a statistical difference exists among patients and controls who had low B12 levels and elevated MMA levels (*p =* 0.012). As we combined both functional biomarkers along with deficient B12 levels, we found that there were four patient and one control cases (Table 3). Combined B12 was also calculated using the algorithms by Fedosov et al. [31], using different biomarkers. We observed a significant difference in the number of patients and controls with low B12 levels using both algorithms (Table 3).

## 4. Discussion

B12 deficiency is multifactorial and can be caused by a decrease in the intrinsic factor, decrease in the digestion of protein-bound B12 from food, or factors like high stress, infections, or antacids, and other medications known to deplete IF [31,32,33,34]. Despite numerous reports on nutritional deficiencies after bariatric surgeries, studies describing B12’s levels and its functional biomarkers among the UAE population are lacking, which prompted us to conduct this present study.

In this study, we report for the first time the levels of B12 along with its functional biomarkers in postoperative LSG Emiratis and compared our findings with a matched control population.

This cross-sectional study shows a higher prevalence of B12 deficiency in patients post LSG compared with matched controls. Specifically, B12 deficiency, defined as a serum level <148 pmol/L, was observed in 28% of patients compared with 5% of controls of similar age, gender, and BMI. The following established functional biomarkers of B12 deficiency were observed: tHcy was found significantly elevated in 55% and 18% of patients and controls, whereas MMA was observed elevated in 28% and 18% of patients and controls, respectively. In concordance with previous studies, our study also infers that B12 deficiency should not be defined solely on its low serum levels, and the abnormal accumulation of functional biomarkers MMA and tHcy should be considered [10,17,22,28,31].

MCA is a potential biomarker that may play a critical role in discerning between metabolic and nutritional B12 deficiencies. Elevated MCA levels have been reported to be associated with B12 metabolic defects [27]. In this study, we did not find any significant differences in the MCA levels between the patient and control groups. Due to tHcy and MMA levels being higher in patients compared with the controls, our results reasonably suggest that MCA may not be an informative biomarker of B12 deficiency in bariatric surgery patients.

In our study, we observed folate deficiency in 28% of patients (<9 nmol/L), which was significantly different in comparison with the control group. B12 deficiency may lead to functional folate deficiency, which is intracellular despite a normal level of folate in the plasma. Folate deficiency may appear after bariatric surgery due to the depletion of tissue stores as a result of inadequate dietary intake and/or impaired absorption following hypochlorhydria and altered intestinal pH. A post-surgery deficiency of folate cycle cofactors, B6, B12, and folate, is associated with increased plasma levels of homocysteine. Folate deficiency after LRYGB surgery has been reported in about 38% of patients and has been attributed to malabsorption and a short bowel [11,35]. The findings of this study are in agreement with previous reports in LRYGB. Therefore, postoperative folate supplementation is recommended either to prevent or correct the folate deficiency occurring due to a compensatory intestinal absorptive capacity [36,37,38]

The optimal therapeutic regimen for B12 supplementation after bariatric surgery needs to be standardized, however, until now, no specific guidelines or recommendations exist [39]. In addition, studies have reported postoperative B12 deficiency that consequently may result in hematologic and neurological damage [16,22]. Though, in the current study, we did not observe hematological or neurologic events, but it cannot be ruled out that some patients eventually may develop megaloblastic anemia or neurologic symptoms if B12 levels remain uncorrected. Indeed, we reasonably consider that a supplementation of B12 to postoperative patients is a prudent strategy to counteract the clinical consequences of subnormal levels of this micronutrient.

The potential limitation of this study is the relatively small sample size and its cross-sectional design. Additionally, it would be interesting to investigate other micronutrients and vitamins to understand the overall nutritional status of the studied population.

## 5. Conclusions

The present study reveals a significant reduction in B12 levels in postoperative LSG patients compared with a matched control population. tHcy and MMA accumulations were detected in the patient group and were statistically significant compared with the control group. This study supports previous observations indicating that tHcy and MMA could be better biomarkers of early changes in the B12 status than serum B12 concentration. Our results suggest that MCA may not be a sensitive marker to detect a nutritional deficiency of B12. To conclude, patients who undergo bariatric surgery should be closely monitored, as they are at a higher risk of B12 deficiency than the normal population.

## Figures and Tables

**Table 1:** Demographic details for 1-year postoperative laparoscopic sleeve gastrectomy (LSG) patients and matched controls.

Variable	Patients (n = 71)	Controls (n = 66)	*p*-Value
Age (years)	36 ± 9	35 ± 10	NS
Female, n (%)	45 (63)	40 (61)	NS
Male, n (%)	26 (37)	26 (39)	NS
Weight (kg)	86 ± 22	82 ± 14	NS
Height (cm)	162 ± 13	159 ± 7	0.009 *
BMI (kg/m^2^)	31 ± 6	32 ± 5	NS
Systolic BP (mmHg)	114 ± 13	119 ± 13	0.011 *
Diastolic BP (mmHg)	70 ± 11	74 ± 8	0.008 *
HbA1c	5.4 ± 0.7	5.6 ± 0.5	NS
Cholesterol	4.9 ± 0.9	4.6 ± 0.6	NS
LDL	3.0 ± 0.8	2.7 ± 0.6	0.054 *
HDL	1.5 ± 0.3	1.0 ± 0.3	<0.000 *
Triglycerides	0.8 ± 0.4	1.0 ± 0.5	0.001 *
Marital status, married	47	43	NS
Marital status, single	24	20	NS
Level of education, educated	70	58	NS
Level of education, illiterate	1	5	NS
Diabetes	0	8	0.003 *
Family history of diabetes	54	42	NS
Hypertension	0	4	0.036 *
Heart Failure	0	0	NS
Dyslipidemia	0	5	0.019 *
Breathlessness	0	7	0.005 *
Sleep apnea	15	9	NS
Gallbladder disease	5	2	NS
Psychological	2	13	0.002 *
Musculoskeletal	0	32	<0.001 *
Clotting abnormality	0	0	NS
Pregnancy complication	0	7	0.005 *
Infertility PCOS	6	9	NS
Fetal defects	0	0	NS
Cancer	0	0	NS

Abbreviations: BMI = body mass index, BP = blood pressure, HbA1c = glycated hemoglobin, HDH = high-density lipoprotein, LDL = low-density lipoprotein, PCOS = polycystic ovary syndrome. * Indicates significant *p* < 0.05.

**Table 2:** Median values for measured metabolic biomarkers in 1-year postoperative LSG patients (n = 71) and matched controls (n = 66).

	Reference Value	Patients Median (Min–Max)	Controls Median (Min–Max)	*p*-Value
B12 (pmol/L)	200–1000	177 (54–907)	334.7 (125.4–1232.5)	<0.001 **
tHcy (µmol/L)	<13.2	13.7 (7.6–29.6)	10.9 (2.9–19.5)	<0.001 **
MMA (µmol/L)	<0.376	0.28 (0.12–2.04)	0.20 (0.08–0.73)	0.011 **
Folate (nmol/L)	>9	11.15 (0.00–36.23)	22.4 (11.3–67.8)	<0.001 **
MCA (µmol/L)	0.021–0.097	0.033 (0.014–0.169)	0.0386 (0.0152–0.0928)	0.266
Hb male (g/L)	130–170	140.5 (116–166)	134 (102–153)	0.005 **
Hb female (g/L)	120–150	115 (70–141)	118 (83–149)	0.043 *

Values are median (range: min–max). Abbreviations: B12 = vitamin B12, Hb = hemoglobin, MCA = methylcitric acid, MMA = methylmalonic acid, tHcy = total homocysteine. (*) *p*-value is less than 0.05; (**) *p*-value is less than 0.01.

**Table 3:** Determination of number of cases with abnormal levels of biomarkers using different approaches among patients (n = 71) and matched controls (n = 66).

	Patients, No. of Cases (%)	Control, No. of Cases (%)	*p*-Value
Using Cutoff by American Society for Metabolic and Bariatric Surgery [9]			
Deficient B12 (<148 pmol/L)	20 (28%)	3 (5%)	<0.001 **
Elevated tHcy (>13.2 µmol/L)	39 (55%)	12 (18%)	<0.001 **
Elevated MMA (>0.376 µmol/L)	20 (28%)	12 (18%)	0.167
Deficient Folate (<9 nmol/L)	21 (30%)	0 (0%)	-
Elevated MCA (>0.097 µmol/L)	2 (3%)	0 (0%)	-
Deficient Hb male (<130 g/L)	3 (3%)	10 (15%)	0.011 *
Deficient Hb female (<120 g/L)	30 (42%)	21 (32%)	0.176
Deficient B12 + Elevated tHcy	10 (14%)	3 (5%)	0.057 *
Deficient B12 + Elevated MMA	9 (13%)	1 (1.5%)	0.012 **
Deficient B12 + Elevated (tHcy + MMA)	4 (5.6%)	1 (1.5%)	0.199
CB12 Calculated using B12 and MMA as Biomarkers [31]			
Elevated CB12 (>1.5)	0 (0%)	0 (0%)	-
CB12 adequacy (−0.5 to1.5)	39 (55%)	57 (86%)	<0.001 **
Low CB12 (−1.5 to −0.5)	27 (38%)	7 (11%)	<0.001 **
Possible CB12(−2.5 to 1.5)	5 (7%)	0 (0%)	-
Probable CB12 deficiency (<−2.5)	0 (0%)	0 (0%)	-
CB12 Calculated using tHcy and MMA as Biomarkers [31]			
Elevated CB12 (>1.5)	0 (0%)	1 (2.1%)	-
CB12 adequacy (−0.5 to 1.5)	27 (38%)	38 (57%)	0.013
Low CB12 (−1.5 to −0.5)	36 (51%)	9 (14%)	<0.001 **
Possible CB12 (−2.5 to −1.5)	7 (9.9%)	0 (0%)	-
Probable CB12 deficiency (<−2.5)	1 (1.4%)	0 (0%)	-

Abbreviations: CB12 = combined vitamin B12, B12 = vitamin B12, Hb = hemoglobin, MCA = methylcitric acid, MMA = methylmalonic acid, tHcy = total homocysteine. (*) p-value is less than 0.05; (**) p-value is less than 0.01

## Supplementary Materials

The following are available online at https://www.mdpi.com/1010-660X/56/3/142/s1, Figure S1: Extracted mass chromatograms of tHcy and d4-Hcy obtained with plasma samples from a control (A) and patient (B). The solid line represents tHcy and dashed line represents the d4-Hcy, Figure S2: Extracted mass chromatograms of MMA and d3-MMA obtained with plasma samples from a control (A) and patient (B). The solid line represents MMA and dashed line represents the d3-MMA, Figure S3: Extracted mass chromatograms of MCA and d3-MCA obtained with plasma samples from a control (A) and patient (B). The solid line represents MCA and dashed line represents the d3-MCA.
