# Supplementary figures and images for: Role of Functional Biomarkers to Identify Early Vitamin B12 Deficiency in Patients with Sleeve Gastrectomy: A Cross-Sectional Study
Source: Medicina (Kaunas). 2020 Mar 20;56(3):142. doi: 10.3390/medicina56030142 (PMC7143905; doi:10.3390/medicina56030142)

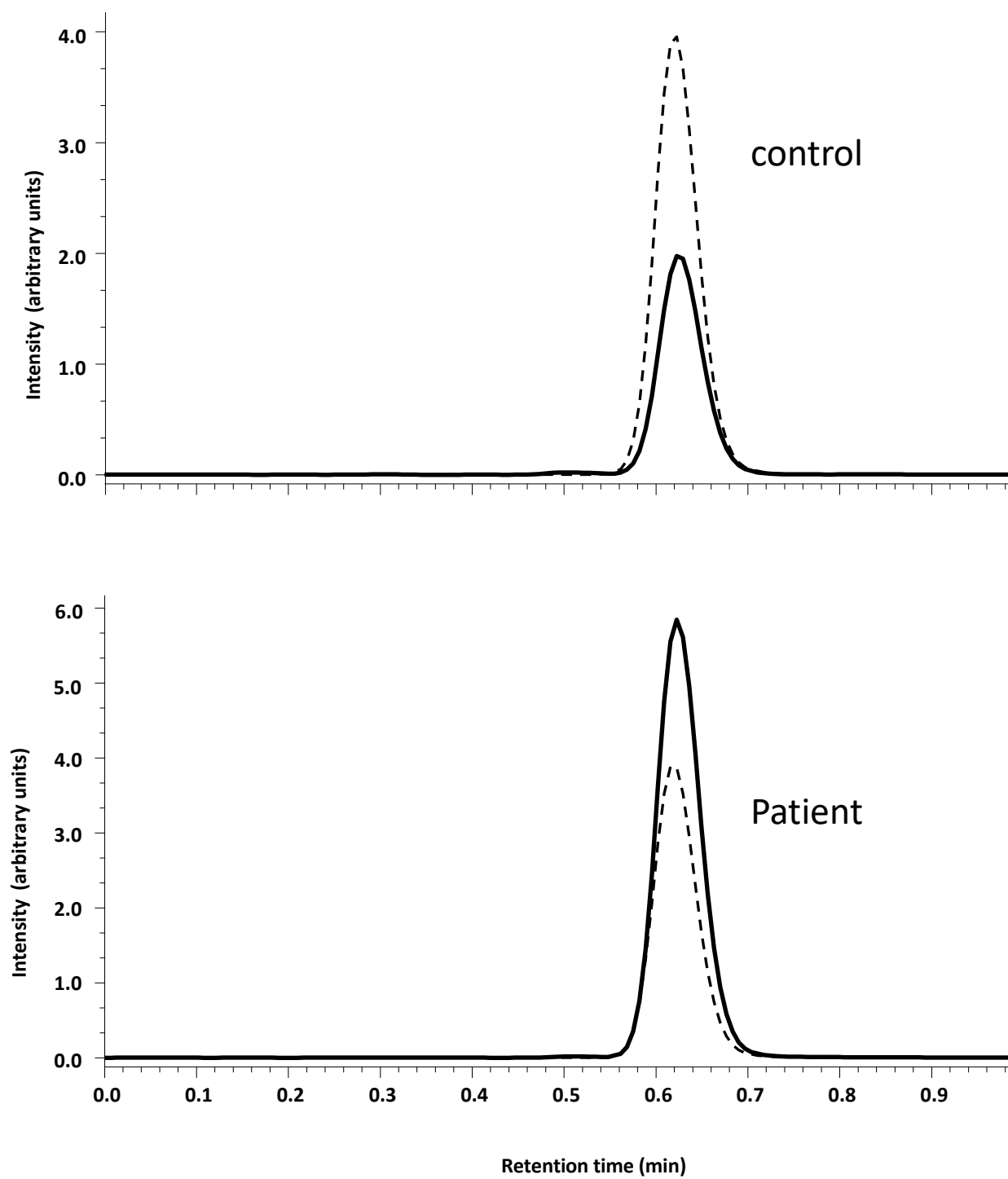

Figure S1

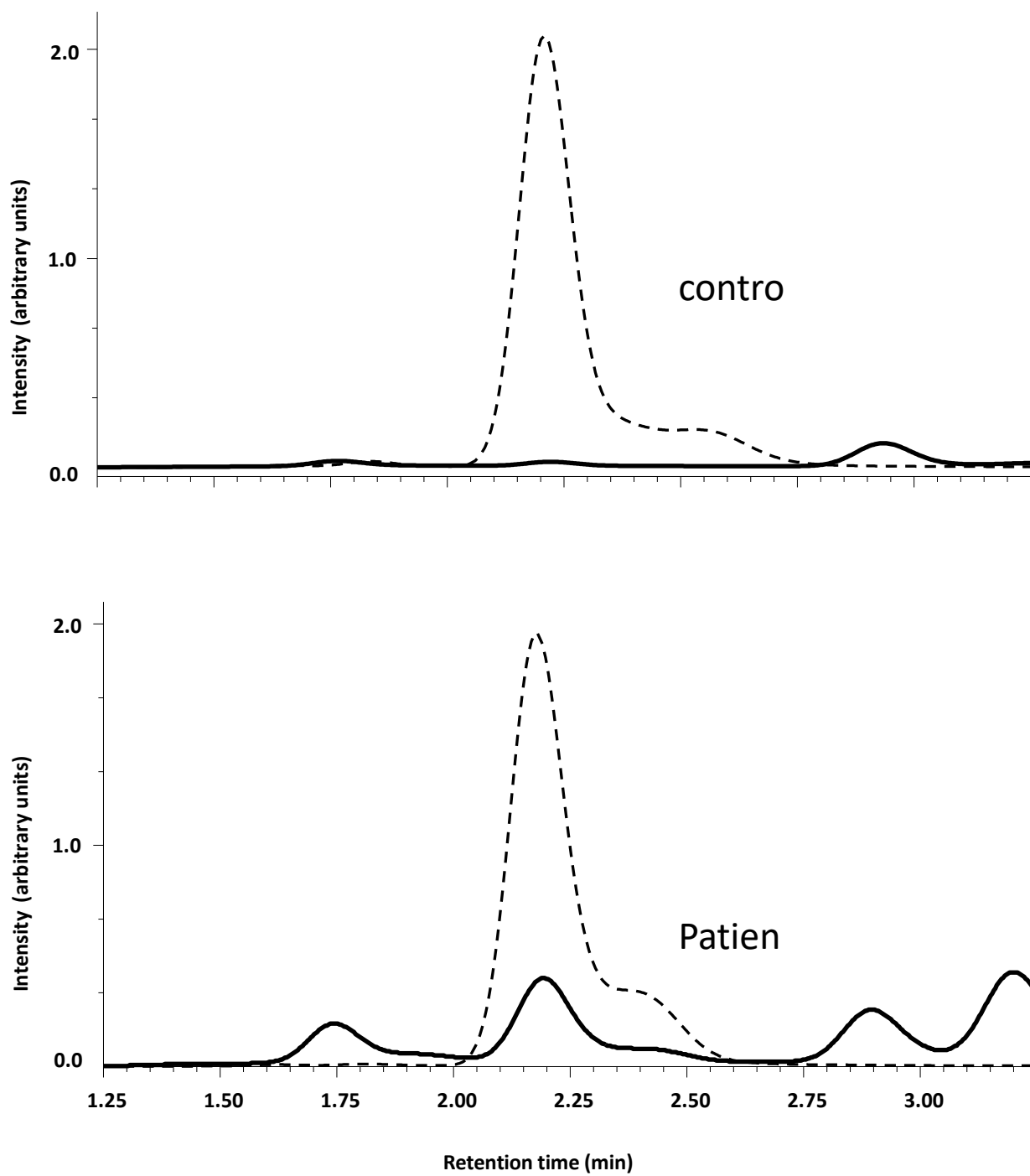

Figure S2

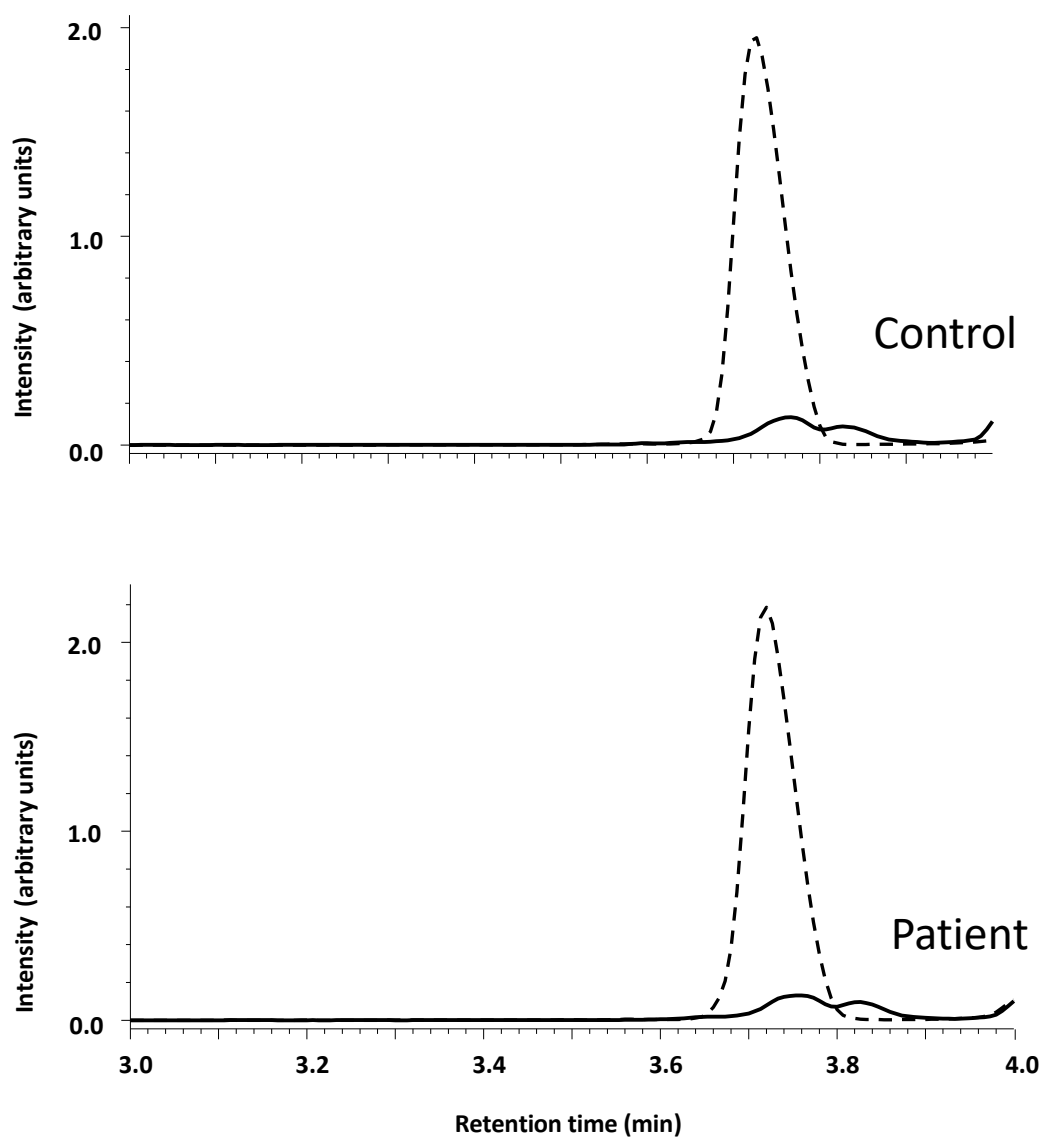

Figure S3

Supplement: Supplementary file 1 [file medicina-56-00142-s001.pdf]
